# A Blend of Old and New: Biomonitoring Methods to Study the Exposome

**DOI:** 10.1289/ehp.125-A74

**Published:** 2017-03-31

**Authors:** Rachel Cernansky

**Affiliations:** Rachel Cernansky is a freelance journalist in Denver, Colorado, covering science, health, and the environment. She has written for publications including *Yale Environment 360*, *Nature*, *Civil Eats*, and *The New York Times*.

The exposome, a concept introduced in 2005, reflects the totality of chemical and nonchemical exposures that an individual accumulates over a lifetime, beginning during prenatal development.^1^ Whereas traditional biomonitoring targets specific analytes to measure in a sample, exposomic approaches include quantifying hundreds or thousands of analytes simultaneously in what is known as untargeted analysis, and measuring an even greater number of metabolites in so-called high-resolution metabolomics. A new commentary in *EHP* discusses why both traditional and exposomic approaches are critical to advancing the science of exposure assessment.^2^

**Figure:**
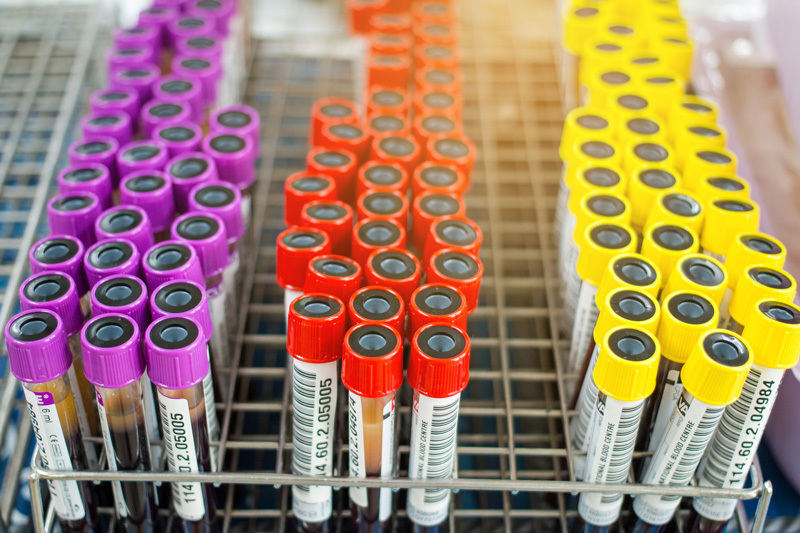
The authors of a new commentary on exposomic research recommend conducting untargeted analyses of samples collected previously for traditional chemical studies. Researchers must also develop methods to detect and identify low-abundance chemicals in samples and to differentiate between endogenous and exogenous molecules. © Krisana Sennok/Shutterstock

The commentary is one paper in a six-part series resulting from a workshop held in January 2015. Coauthor David Balshaw, chief of the Exposure, Response, and Technology Branch at the National Institute of Environmental Health Sciences (NIEHS), says that environmental health scientists are increasingly aware of the idea of the exposome, but that some view it with some skepticism—both because of concerns about its untargeted hypothesis-generating approach and because existing technologies are still catching up to the concept of measuring the exposome.

“What this series is intended to do is address those concerns—and to say that [exposomics] is still an emerging concept that needs additional capability and additional validation,” Balshaw says. “As I view the exposome and similar untargeted approaches, they are a tool for hypothesis generation. They do not replace the scientific method; they supplement it.”

The authors identified gaps in existing biomonitoring technology, which formed the basis of eight recommendations discussed in the commentary for advancing exposomic research. Among these recommendations are conducting untargeted analyses of samples collected previously for traditional targeted chemical studies, creating tools to search across multiple complementary databases, and developing chemistry methods to detect low-abundance chemicals and differentiate between endogenous and exogenous molecules among the thousands measured in an untargeted analysis.

A fourth recommendation is developing bioinformatics techniques to enhance detection of unknown chemicals. Balshaw points to coauthor Gary Patti’s work on untargeted metabolomics as an example of how the recommendations build on existing work. In a 2012 study^3^ Patti and colleagues described a way to more efficiently identify metabolites detected through untargeted studies. The authors came up with a database and workflow to automate the processing of the voluminous data produced by such studies.

Looking ahead, Balshaw says the NIEHS-led Children’s Health Exposure Analysis Resource (CHEAR)^4^ will focus on developing infrastructure to help realize the recommendations in the commentary. Among other services, CHEAR conducts both targeted and untargeted analyses of biosamples collected by children’s health researchers funded by the National Institutes of Health (NIH).

Robert Wright, director of the Lautenberg Laboratory for Environmental Health at Mount Sinai’s Icahn School of Medicine, thinks the discussion of current data-processing capacity is the commentary’s key contribution. “This is probably the best paper I’ve ever read in terms of detailing how to measure the exposomics assays, and laying out the bioinformatics challenges as well,” he says. “I think people get caught up in the technology of measuring the assay, but you have to do something with [the data produced].” Wright was not involved in the commentary.

For Tracey Woodruff, director of the Program on Reproductive Health and the Environment at the University of California, San Francisco, another important consideration is the potential to identify chemical signatures—unique patterns of changes in molecules associated with a particular exposure or health end point—which she sees as a crucial link between research and application. “If our goal is to improve health, then we have to figure out [which signatures] are bad,” she says. Woodruff was not involved with the commentary.

Woodruff is encouraged by the emphasis on untargeted studies. To her, the commentary is an important indication that NIH is looking to include more hypothesis-generating research in its funding portfolio—as opposed to projects using the traditional approach of starting with a particular hypothesis, which, like targeted studies, is more limiting.

“Post-its weren’t created because someone was trying to create a Post-it. They were invented because someone was trying to make glue for something else. We’re trying to find a new Post-it,” Woodruff says. “That NIH is saying we want to see more of this type of broad research in the field is very, very important. It represents a commitment to a shift in the type of research that’s being funded and ultimately toward supporting efforts to identify environmental contributors to disease.”
